# Arginine and Lysine Supplementation Potentiates the Beneficial β-Hydroxy ß-Methyl Butyrate (HMB) Effects on Skeletal Muscle in a Rat Model of Diabetes

**DOI:** 10.3390/nu15224706

**Published:** 2023-11-07

**Authors:** Manuel Manzano, María D. Girón, Rafael Salto, Chiara Burgio, Antonio Reinoso, Elena Cabrera, Ricardo Rueda, Jose M. López-Pedrosa

**Affiliations:** 1 Abbott Nutrition R&D, E18004 Granada, Spain; manuel.manzano@abbott.com (M.M.); ricardo.rueda@abbott.com (R.R.); jose.m.lopez@abbott.com (J.M.L.-P.); 2 Department of Biochemistry and Molecular Biology II, School of Pharmacy, University of Granada, E18071 Granada, Spain; mgiron@ugr.es (M.D.G.); chiaraburgio@ugr.es (C.B.); areinoso@ugr.es (A.R.); elenacc202@gmail.com (E.C.)

**Keywords:** β-hydroxy β-methyl butyrate, arginine, lysine, diabetes, muscle, glucose uptake, protein synthesis, lean body mass

## Abstract

Skeletal muscle is the key tissue for maintaining protein and glucose homeostasis, having a profound impact on the development of diabetes. Diabetes causes deleterious changes in terms of loss of muscle mass, which will contribute to reduced glucose uptake and therefore progression of the disease. Nutritional approaches in diabetes have been directed to increase muscle glucose uptake, and improving protein turnover has been at least partially an oversight. In muscle, β-hydroxy β-methyl butyrate (HMB) promotes net protein synthesis, while arginine and lysine increase glucose uptake, albeit their effects on promoting protein synthesis are limited. This study evaluates if the combination of HMB, lysine, and arginine could prevent the loss of muscle mass and function, reducing the progression of diabetes. Therefore, the combination of these ingredients was tested in vitro and in vivo. In muscle cell cultures, the supplementation enhances glucose uptake and net protein synthesis due to an increase in the amount of GLUT4 transporter and stimulation of the insulin-dependent signaling pathway involving IRS-1 and Akt. In vivo, using a rat model of diabetes, the supplementation increases lean body mass and insulin sensitivity and decreases blood glucose and serum glycosylated hemoglobin. In treated animals, an increase in GLUT4, creatine kinase, and Akt phosphorylation was detected, demonstrating the synergic effects of the three ingredients. Our findings showed that nutritional formulations based on the combination of HMB, lysine, and arginine are effective, not only to control blood glucose levels but also to prevent skeletal muscle atrophy associated with the progression of diabetes.

## 1. Introduction

Muscle plays a significant role in glucose homeostasis, making it a key target in the pathophysiology of type 2 diabetes mellitus (T2DM). Skeletal muscle is responsible for 80% of postprandial glucose clearance from circulation, and it is considered the primary driver tissue that contributes to whole-body insulin-mediated glucose disposal [1]. Muscle atrophy is a common complication of diabetes, characterized by a reduction in muscle mass and strength that contributes to decreased mobility, functional impairment, and increased mortality risk [2,3]. In addition, in the Korean Sarcopenic Obesity Study (KSOS), which examined the prevalence of sarcopenia in 810 subjects (396 well-functioning community-dwelling healthy subjects and 414 patients with T2DM) [4], older patients with T2DM had a three times higher risk of sarcopenia than subjects without diabetes after adjusting for potential risk factors. This has also been reported by other studies [2,5,6,7,8].

Muscle loss in diabetes is a multifactorial process involving several mechanisms, such as altered protein metabolism, mitochondrial dysfunction, oxidative stress, inflammation, and insulin resistance [9]. Sarcopenia and diabetes act synergically, leading to a vicious cycle that exacerbates muscle loss and impairs muscle function [10]. For instance, insulin resistance, which is defined as a reduced target tissue response to insulin, reduces muscle protein synthesis and increases protein breakdown, resulting in a loss of muscle mass. Consequently, this muscle dysfunction aggravates the course of T2DM and sarcopenia at the same time [11].

There is a need to conserve skeletal muscle in people with diabetes particularly to reduce insulin resistance and ultimately to break the vicious cycle that accelerates the progression of diabetes-associated complications. Pharmacological and lifestyle modifications have been proposed as strategies for preserving skeletal muscle mass and function in diabetes [7]. Glucose-lowering drugs, such as insulin [12], metformin [13], and thiazolidinediones [14], improve insulin sensitivity and might have a beneficial effect on parameters of sarcopenia. Lifestyle modifications, such as weight loss and dietary changes, have been shown to improve insulin sensitivity and muscle function. Physical exercise, particularly resistance training, enhances muscle mass and strength, improves glucose uptake and utilization, and reduces inflammation and oxidative stress. Nutritional intervention seems also to be a key strategy, not only for managing muscle mass and strength but also for preventing the progression of diabetes. However, to the best of our knowledge, there are few studies in which muscle loss and glycemic control are simultaneously addressed through nutritional intervention strategies.

β-hydroxy-β-methyl butyrate (HMB) is a metabolite of leucine that has been shown to reduce muscle catabolism, enhance muscle protein synthesis, and improve muscle function [15]; however, its serum levels are reduced during aging [16]. The role of HMB supplementation in the regulation of glucose homeostasis remains unclear. While there are studies that describe a positive effect of HMB supplementation on glycemic control and other parameters related to diabetes [17,18], other studies have described conflicting effects on insulin sensitivity [19,20,21].

Supplementation with lysine (Lys) and arginine (Arg) has shown beneficial effects on diabetes. Lys is an essential amino acid. Regarding the effect of Lys supplementation in diabetes, numerous beneficial effects have been demonstrated in the treatment/prevention of the disease and/or its complications, both in animal studies and clinical studies [22,23,24]. Arg is considered a conditionally essential amino acid because it cannot be adequately synthesized by infants or adults [25]; it has been shown to have a positive role in the control of diabetes. There are several lines of evidence, supported by preclinical and clinical studies, indicating that Arg might have beneficial effects for the treatment of diabetes. Arg is a powerful secretagogue that regulates insulin [26,27] and glucagon secretion [28]. Arg supplementation has also been shown to improve glucose tolerance and improve insulin sensitivity, as well as reduce adiposity [28].

Considering the above, the objective of the current study was to determine if a combination of HMB, Lys, and Arg might have an effect on blood glucose control and could counteract the muscle mass and function loss observed in diabetes conditions, using preclinical in vitro and animal models that mimic the metabolic alterations found in T2DM. Our findings showed that nutritional formulations based on the combination of HMB, Lys, and Arg might result in being useful, not only in controlling blood glucose levels but also in preventing skeletal muscle atrophy associated with diabetes progression.

## 2. Materials and Methods

### 2.1. Cells Culture

The L6.C11 rat skeletal muscle myoblast line (ECACC No. 92102119) was grown in DMEM supplemented with 10% (*v*/*v*) FBS, 2 mmol/L glutamine plus 100 units/mL penicillin, and 0.1 mg/mL streptomycin in an atmosphere of 5% CO_2_ and 95% air. It was maintained at sub-confluent densities in the growth media. Cells were differentiated into myotubes by culturing them for 5 days in DMEM containing 2% FBS (*v*/*v*).

The cells were incubated in the absence or presence of Arg (0–20 mM), Lys (0–20 mM), or HMB (0–50 µM). Some experiments were performed in cells incubated in the presence of 0.5 mM palmitate-BSA, a well-known model of insulin resistance in skeletal muscle cells [29].

### 2.2. 2-Deoxy-[3H]D-glucose Uptake

Cells were grown in 48-well plates (Corning, NY, USA). They were incubated with different effectors overnight. Then, cells were rinsed with KRPH (HEPES-buffered Krebs–Ringer phosphate), consisting of 118 mmol/L NaCl, 5 mmol/L KCl, 1.3 mmol/L CaCl_2_, 1.2 mmol/L MgSO_4_, 1.2 mmol/L KH_2_PO_4_, and 30 mmol/L HEPES (pH 7.4). The 10 μmol/L 2-deoxy-[^3^H]D-glucose (2-DG) (1 μCi/mL) uptake was measured over a 10-minute period under conditions in which the uptake was linear. The uptake measurement was made in triplicate. The uptake of 2-DG was ended after 10 min by rapidly aspirating off the radioactive incubation medium and washing the cells three times in ice-cold phosphate-buffered saline. The radioactivity associated with the cells was determined by cell lysis in 0.5 N NaOH, with neutralization by the addition of 0.5 N HCl, followed by liquid scintillation. Aliquots from each well were used to determine the protein concentration, using the BCA Protein assay.

### 2.3. Determination of Protein Synthesis and Degradation

Protein synthesis was measured as described [30], with some modifications [31,32]. Data were computed as dpm/µg of proteins.

For the measurement of protein degradation, myotubes (5 days in differentiation media) were labeled with 1 μCi/mL of L-[ring-3, 5–^3^H]-tyrosine (Tyr) for 48 h in DMEM plus 10% FBS in the presence or absence of the different effectors. Cells were rinsed once in PBS-Tyr and then placed in DMEM supplemented with 10% FBS and 2 mM L-tyrosine plus 5 μM DEX (degradation medium) for 2 h to allow for the degradation of very short-lived proteins. The cells were then rinsed twice in PBS-Tyr, and a fresh degradation medium was added. Cells were incubated for an additional 24 h in a degradation medium. Degradation rates were determined as previously described [31,32].

For ubiquitin gene reporter analysis, cells were used at 80–90% confluence. Transfection was performed using LipofectAMINE2000 as described by the manufacturer. The DNA mixture comprised the pGL3-UbC luciferase reporter and the reference plasmid pRL-TK (ratio 95:5) [31].

Cells were then incubated in the presence or absence of 5 μM DEX or 0.5 mM palmitate-BSA for 24 h in combination with the effectors. Luciferase activity was determined using the Dual-Luciferase method (Promega, Alcobendas, Madrid, Spain) in a luminometer (Sirius L, Berthold Technologies, Bad Wildbad, Germany), and the results were standardized for Renilla luciferase activity. To allow for a comparison of the expression patterns, the data are expressed as relative changes in luciferase activity and were normalized to a value of 100%.

### 2.4. Plasma Membrane Isolation

A plasma membrane-enriched fraction from myotubes was prepared as described [33]. Caveolin expression was used as a marker for plasma membrane enrichment.

### 2.5. Protein Analysis

To study the expression and phosphorylation status of the proteins involved in metabolic and signaling events, L6 myotubes were incubated in the absence or presence of effectors for 30 min, 2 h, or 24 h. Plates were flash-frozen in liquid nitrogen and processed as described previously [31,33].

The protein concentration was measured using the bicinchoninic acid method [34]. Proteins (40 μg) were separated by SDS-PAGE, transferred onto nitrocellulose membranes, and immunoblotted with specific antibodies. To assay the phosphorylation degree of key kinases, antibodies against phosphorylated proteins were used. To determine the expression of other proteins, the amount of GAPDH was used as a load control. The immunoblots were developed by using fluorescent secondary antibodies (Bio-Rad, Madrid, Spain).

### 2.6. Animals’ Conditions and Acclimatization

Male Wistar Han rats (11–13 weeks old) were provided by Envigo (France). Animals were individually housed in cages and kept under 12 h light–12 h dark cycles. The room temperature was maintained at 21 °C.

All experimental procedures (approval codes 14 July 2020/080 and 15 March 2022/039) were carried out according to the European Convention for the Protection of Vertebrate Animals used for Experimental and other Scientific Purposes (Directive 2010/63/EU), as well as to the ethical guidelines for animal experimentation provided by the Spanish National Research Council (RD 53/1 February 2013).

### 2.7. Animal Model of Insulin Resistance: Euglycemic–Hyperinsulinemic Clamp

A high-fat diet was utilized to produce a model of insulin resistance, where all the rats used, except for the control lean rats’ group (AIN93M), were subjected to a 42% total calories high-fat diet (Supplementary Materials Table S1) (HF) [35] throughout the study. Rats fed the HF diet were divided into two groups, depending on whether the diet was supplemented with HMB (0.78%; HF-H group) or not (HF group).

After 28 days of nutritional treatment, the insulin-sensitizing effect of HMB was evaluated using a modified euglycemic–hyperinsulinemic clamp technique developed by De Fronzo et al. [36]. In brief, 12 hour-fasted rats were anesthetized with an intraperitoneal injection of sodium pentobarbital (50 mg/kg bw). A tracheostomy was performed to facilitate tracheal clearing. Two catheters were placed in the right jugular vein for glucose and insulin infusion. Another catheter was placed in the left carotid artery for blood sampling. After approximately 30 min of surgery, the basal blood glucose levels of the rats were detected. At time 0, human insulin (Humulin R, Eli Lilly, IN) was infused at a concentration of 15 mU/kg per minute. Blood samples were subsequently drawn at five-minute intervals for the determination of blood glucose (glucometer). An infusion of 30% glucose was adjusted to maintain blood glucose at 100 mg/dL. A steady state was ascertained when a fixed glucose infusion rate kept the blood glucose measurements constant for at least 30 min.

### 2.8. Animal Model of Diabetes: Assessment of Muscle Mass and Metabolism

In a second animal model, a high-fat diet/low streptozotocin dose method was utilized to induce type 2 diabetes mellitus (T2DM) in rats [37]. Animals were divided into four nutritional groups (Supplementary Materials Table S2). Rats were fed an HF diet (HF/STZ group); an HF diet supplemented with HMB, Lys, and Arg (HF+HAas/STZ group); an HF diet whose carbohydrates were replaced by a mixture of slow digestive carbohydrates (HF+SDC/STZ group); or an HF diet with the mixture of slow digestible carbohydrates supplemented with HMB, Lys, and Arg (HF+HAas+SDC/STZ group) throughout the study. The diabetes was induced by the administration of an intraperitoneal single low dose of streptozotocin (STZ, 30 mg/kg body weight) to deplete pancreatic beta cells. The nutritional treatment was initiated on the 42 days pre-STZ administration, which was considered the first day of the regimen and continued thereafter until the end of the study.

All rats had free access to food and water, and animal weight and food consumption were determined weekly. Soft lean body mass was measured before the STZ injection and at the end of the study by quantitative nuclear magnetic resonance imaging (EchoMRI 700 system; Echo Medical Systems, Houston, TX, USA).

At the end of the study, blood was collected and glucose, HbA1c, triacylglycerols (TGs), cholesterol, low-density lipoprotein (LDL) cholesterol, and high-density lipoprotein (HDL) cholesterol were analyzed using a clinical chemistry analyzer, Pentra 400 (Horiba ABX, Montpellier, France). Muscle samples were used for the study of the expression and phosphorylation status of the proteins involved in metabolic and signaling events as described above.

### 2.9. Statistical Analysis

Results were expressed as means ± SEM. Statistical significance was set up at *p* < 0.05. Statistical significance between groups was determined using Student’s *t*-test.

## 3. Results

### 3.1. HMB Effects on Glucose Uptake and Protein Turnover in L6 Cells in Culture

**Figure 1:**
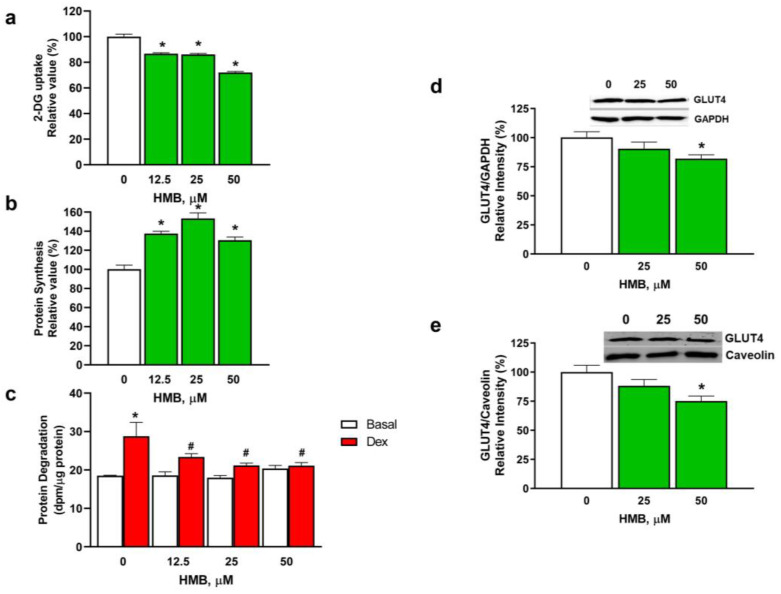
Effects of HMB on glucose uptake and protein turnover in L6 myotubes. L6 myotubes were incubated with HMB (0–50 µM) for 24 h, and 2-DG uptake (**a**) and protein synthesis (**b**) were measured. Protein degradation was measured (**c**) in the absence or presence of 5 μM dexamethasone (Dex). GLUT4 expression was determined in cell lysates (**d**) or plasma membranes (**e**) via Western blot in myotubes incubated for 24 h (**d**) or 2 h (**e**). Data are expressed as mean ± SEM (*n* = 8). * *p* < 0.05 vs. cells incubated in the absence of effectors; # *p* < 0.05 vs. Dex incubated cells.

To address the effects of HMB on glucose uptake in skeletal muscle cells in culture, we carried out experiments where increasing doses of HMB were assayed in differentiated L6 myotubes. As shown in Figure 1a, HMB produced a moderate but significant decrease in glucose uptake at all the ranges of doses used. On the contrary, at these doses, a significant increase in net protein synthesis was detected (Figure 1b), and a decrease in protein degradation (induced by pre-incubation with dexamethasone) was obtained (Figure 1c). Therefore, although HMB from 12.5 to 50 µM can enhance protein turnover in muscle cell cultures, it has a limited effect on glucose uptake.

To further analyze the effects of HMB on glucose uptake, 25 and 50 µM were selected, and their effects on the total amount of GLUT4 transporter and the GLUT4 plasma membrane fraction were analyzed via a Western blot (Figure 1d,e). Our results indicate that, at the higher concentration assayed, a significant decrease in the total and plasma-associated amounts of the transporter was detected.

To identify the molecular bases of the effects of HMB on glucose uptake and GLUT4 expression, experiments directed at assaying the status of the insulin signaling pathway were carried out. First, the phosphorylation of IRS-1 was analyzed for several residues (Supplementary Materials Figure S1a). Our data show that HMB produced a significant increase in the phosphorylation of IRS-1 at Ser302, which is responsible for the activation of the pathway. However, this increase was significantly lower in cells incubated with 50 µM HMB than in 25 µM. Furthermore, when phosphorylation at Ser636/639 and Ser1011 (that have been described as downregulators of the downstream signaling) was analyzed, a significant increase in the phosphorylation of these positions was detected at 50 µM HMB. In agreement with these results, 25 µM HMB produced a significant increase in the phosphorylation of the Akt kinase, while 50 µM HMB partially blocked the activation of the kinase (Supplementary Materials Figure S1b). Since the translocation of GLUT4 to the plasma membrane decreased upon incubation with the higher doses of HMB, the activity of AS160, a key protein that regulates GLUT4 translocation, was studied. For this, first, cells were incubated with okadaic acid, a well-known inhibitor of protein phosphatases. Our results (Supplementary Materials Figure S1c) indicate that the incubation with okadaic acid can rescue the decrease in glucose uptake at the HMB concentrations assayed, pointing out that the effects of HMB could be due to a decrease in the phosphorylation status of the AS160 protein. When the phosphorylation of this protein at the residues that activate GLUT4 translocation to the plasma protein in the presence of HMB was analyzed, no significant changes in the phosphorylation of the kinase were detected (Supplementary Materials Figure S1d).

### 3.2. HMB Supplementation in an Animal Model of Insulin Resistance Has a Neutral Effect on Insulin Sensitivity

**Figure 2:**
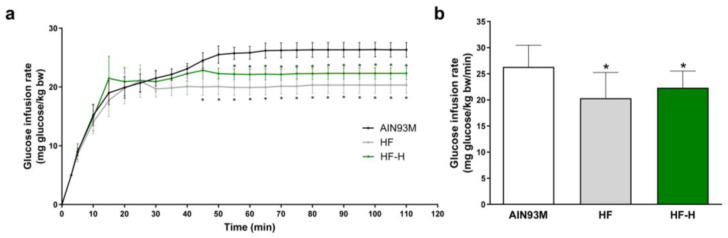
HMB effects on whole-body insulin sensitivity in a high-fat model of insulin resistance. Rats were fed a low- (AIN93M) or high-fat diet that was supplemented (HF-H) or not (HF) with HMB for four weeks, and the insulin sensitivity was measured using a modified euglycemic–hyperinsulinemic clamp. (**a**) Time course of the clamp. (**b**) Glucose infusion rates. Data are expressed as mean ± SEM (*n* = 10). * *p* < 0.05 vs. AIN93M diet.

To extrapolate the cell culture results to animal models, a preliminary experiment to test the effects of HMB supplementation on insulin sensitivity was carried out in a high-fat rat model of insulin resistance. In this model, animals were fed a low or high-fat diet supplemented or not with HMB for four weeks. Then, their insulin sensitivity was measured by a modified euglycemic–hyperinsulinemic clamp technique [36], and the results are shown in Figure 2.

The results indicate, as expected, that an HF diet translated to insulin resistance. More relevant, when the HF diet was supplemented with HMB, no significant differences were found with the group fed the HF diet.

### 3.3. Amino Acids Supplementation Could Ameliorate the HMB-Induced Decrease in Glucose Uptake in L6 Cells

**Figure 3:**
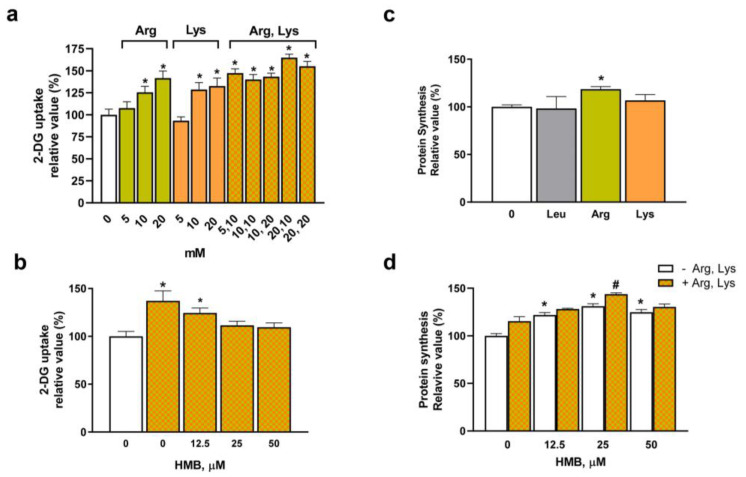
HMB supplementation with amino acid supplementation improves glucose uptake and protein synthesis in L6 cells. The uptake of 2DG was analyzed in L6 myotubes incubated with 5–20 mM Arg or Lys and their combinations (**a**) or in cells supplemented with 0–50 µM HMB plus 5 mM Arg and 10 mM Lys (**b**). Protein synthesis was measured in myotubes incubated in the presence of 10 mM amino acids (**c**) or with 0–50 µM HMB plus 5 mM Arg and 10 mM Lys (**d**). Data are expressed as mean ± SEM (*n* = 8). * *p* < 0.05 vs. cells incubated in the absence of effectors; # *p* < 0.05 vs. 25 µM HMB incubated cells.

To potentiate the effects of HMB on glucose uptake while maintaining its positive effects on protein turnover, we considered the possibility of supplementation with specific amino acids. For that purpose, first, we assayed the effects on the glucose uptake of increasing concentrations of two amino acids, Lys and Arg, alone (Figure 3a). Since supplementation with both amino acids has a significant effect on glucose uptake in the muscle cell cultures at 10 and 20 mM, several combinations of them were tested for glucose uptake (Figure 3a). Our results indicate that, for all the concentrations tested, the combination of amino acids enhances glucose uptake, and therefore the smaller concentration (10 mM Lys and 5 mM Arg) was selected for further analysis.

The effects of the 10 mM Lys and 5 mM Arg supplementation in the presence of HMB on glucose uptake were analyzed. For that, cells were incubated with increasing amounts of HMB plus the amino acid mixture (Figure 3b). The results indicate that supplementation with the amino acids, in the range of HMB concentrations from 12.5 to 50 µM, can ameliorate the effects of HMB on glucose uptake, and, at 12.5 µM HMB plus amino acids, a significant increase in 2-DG uptake was detected.

Next, the individual effects of the amino acids, as well as the combination of them in the presence or absence of HMB on protein synthesis, were tested. First (Figure 3c), the individual effects of both amino acids were assayed at 10 mM concentrations. Leu was included as a negative control (since in muscle cells is HMB and not Leu the molecule that can promote protein synthesis [32]). Our results show that neither Leu nor Lys has any significant effect on promoting protein synthesis, while Arg mediates a moderate increase in protein synthesis. More interestingly, when the combination of amino acids was assayed in the presence of increasing concentrations of HMB, an additive effect of the mixture of amino acids on protein synthesis was detected at 25 µM HMB (Figure 3d).

Therefore, HMB, when combined with Lys and Arg, has a positive effect on glucose uptake in muscle cell culture. Under these conditions, the positive effects of HMB on protein synthesis remain in place.

**Figure 4:**
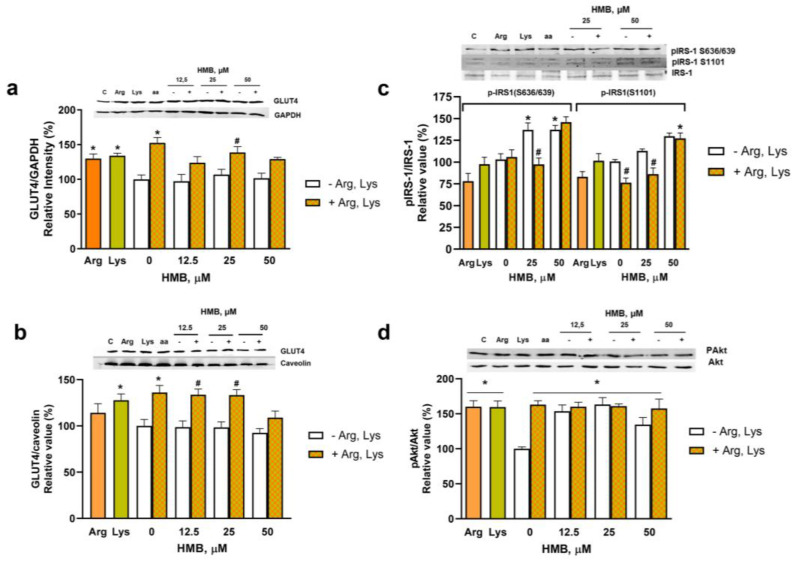
Effects of the supplementation with HMB, Lys, and Arg on key parameters involved in the muscle cell glucose uptake. L6 myotubes were incubated with 10 mM Lys alone, 5 mM Arg alone, or both in combination with HMB (0–50 µM) for 24 h, and GLUT4 expression was measured in cell lysates (**a**) or an enriched plasma membrane fraction (**b**). In (**c**,**d**), cells were incubated with the same effectors as in (**a**) for 30 min, and the phosphorylation of IRS-1 in residues responsible for the insulin resistance and of Akt was analyzed. Data are expressed as mean ± SEM (*n* = 4). * *p* < 0.05 vs. cells incubated in the absence of effectors; # *p* < 0.05 vs. HMB incubated cells.

Next, we analyzed the effects of the supplementation with the amino acids in the presence or absence of HMB on key parameters involved in muscle glucose uptake (Figure 4). First, the effect of individual amino acids and their combination was assayed on the total amount of GLUT4 in cell lysates. As expected, and in agreement with the glucose uptake data, Lys and the combination of the two amino acids can significantly increase the total amount of the transporter. The increase in GLUT4 levels remained in the presence of 25 µM HMB (Figure 4a). Furthermore, when the expression of GLUT4 at the plasma membrane was assayed, the supplementation with the mixture of amino acids produced a significant increase in GLUT4 translocation to the plasma membrane in the cells incubated with up to 25 µM HMB (Figure 4b). These results justify the positive effect of the three ingredients’ combination on glucose uptake.

Next, the phosphorylation status of IRS-1 at the residues that produced the inhibition of insulin signaling was analyzed. Our results (Figure 4c) indicated that the phosphorylation mediated by HMB at these residues decreased upon incubation with the amino acid mixture, and, therefore, the concomitant Akt phosphorylation was maintained by the amino acid mixture in the presence or absence of HMB (Figure 4d).

### 3.4. Amino Acid Supplementation Retains Its Positive Effects on Glucose Uptake in L6 Cells in Conditions That Produce Insulin Resistance

**Figure 5:**
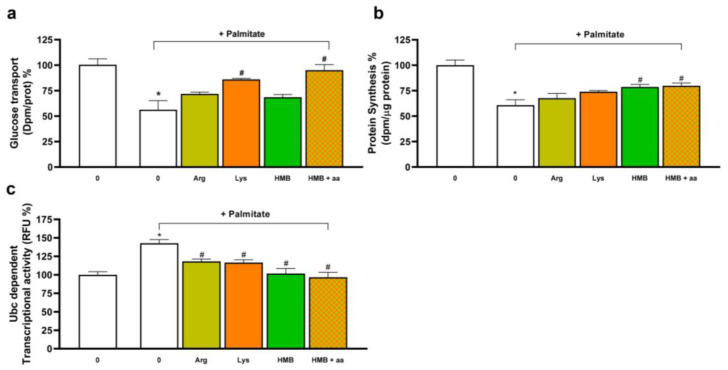
Amino acid supplementation retains its positive effects on glucose uptake and protein turnover in L6 cells in an in vitro insulin-resistance model. L6 myotubes were incubated with 10 mM Lys alone, 5 mM Arg alone, or both in combination with 25 µM HMB for 48 h in the absence or presence of 0.5 mM palmitate-BSA. Uptake of 2-DG (**a**), protein synthesis (**b**), or UbC promoter-dependent transcriptional activity (**c**) was measured. Data are expressed as mean ± SEM (*n* = 4). * *p* < 0.05 vs. cells incubated in the absence of effectors; # *p* < 0.05 vs. palmitate incubated cells.

Since our objective was to implement the use of HMB in insulin-resistance situations, the effects of HMB and the supplementation with amino acids were assayed in a well-known cell culture model of insulin resistance [29]. In Figure 5, the results obtained in L6 muscle cells incubated in the presence of palmitate are shown. Pre-incubation of the cells with palmitate constitutes a model to induce insulin resistance and decrease glucose uptake in these cells, as shown in Figure 5a. More interesting, under these conditions that decrease glucose uptake, supplementation with the individual amino acids, as well as the incubation of HMB in the presence of the amino acids’ mixture, was able to significantly increase glucose uptake.

Furthermore, the effects of the amino acid supplementation on protein synthesis were assayed in this model. Although the incubation with palmitate significantly decreased the net protein synthesis, supplementation with HMB and the combination of HMB plus the amino acids was able to increase the synthesis compared to the palmitate-treated cells. In addition, when the effects of the supplementation with HMB or the combination of HMB plus amino acids were assayed using the ubiquitin (UbC) dependent transcriptional activity as a reflection of the increased protein degradation in the muscle cells pre-incubated with palmitate, our results (Figure 5c) indicated that, while pre-incubation with palmitate was able to stimulate the transcription dependence of the ubiquitin promoter, all the treatments that were assayed significantly decreased this transcriptional activity, pointing out to a normalization of the protein turnover in these conditions.

### 3.5. HMB and Amino Acids Supplementations in an Animal Model of Diabetes Improve Glucose Uptake and Lean Body Mass

To validate the use of HMB and the amino acids mixture, a rat model of diabetes was used. This type 2 diabetes model is based on the use of a high-fat diet and the administration of streptozotocin [37]. To use this model, animals were fed the different experimental diets (Supplementary Materials Table S2), and the diabetes was induced via an STZ injection. All experimental groups were fed an HF diet supplemented either with a rapid digestive carbohydrate mixture (HF/STZ) or with a rapid digestive carbohydrate with HMB, Lys, and Arg (HF+HAas/STZ), or with slow digestive carbohydrates in the absence (HF+SDC/STZ) or presence of HMB, Lys, and Arg (HF+HAas+SDC/STZ). The body weights and food intake of the different groups are indicated in Supplementary Materials Table S3.

**Figure 6:**
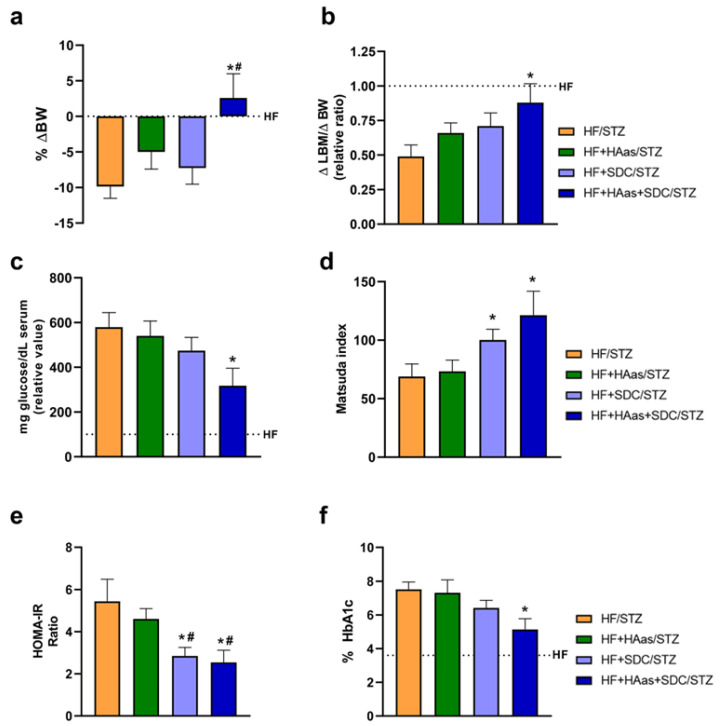
Effects of the supplementation with HMB, Lys, and Arg on lean body mass and glucose homeostasis in HF/STZ rats. A high-fat diet/low streptozotocin dose method was utilized to induce type 2 diabetes mellitus in rats. Rats were fed with the HF diet supplemented with HMB, Lys, and Arg (HAas), with or without slow digestive carbohydrates (SDCs), for one month. Changes in body weight (BW) (**a**), lean body mass (LBM) (**b**), fasting glucose (**c**), insulin sensitivity (**d**), HOMA-IR (**e**), and fasting HbA1c (**f**) are shown. Rats fed an HF diet before the STZ injection were considered the reference group. Data are expressed as mean ± SEM (*n* = 10). * *p* < 0.05 vs. rats fed the HF/STZ diet; # *p* < 0.05 vs. HF+HAas/STZ rats.

At the end of the experiment, a significant increase in body weight was detected in the HF+HAas+SDC/STZ group, compared to the other experimental groups (Figure 6a). More interestingly, the increase in body weight matches an increase in lean body mass in this experimental group.

The HF+HAas+SDC/STZ group presented a significant decrease in glycemia (Figure 6c) associated with higher insulin sensitivity (Figure 6d,e). Furthermore, this experimental group showed a decrease in glycated hemoglobin compared to the other experimental groups (Figure 6f). Additionally, the cardiovascular risk was analyzed in these groups. For that, triacylglycerols and cholesterol levels were measured (Supplementary Materials Figure S2). Again, the HF+HAas+SDC/STZ group showed a significant decrease in cardiovascular risk compared to the other experimental groups.

**Figure 7:**
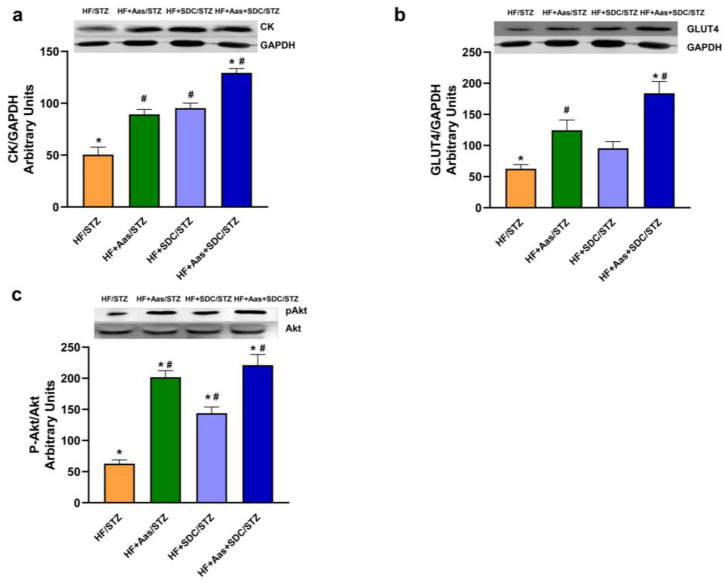
Effects of the supplementation with HMB, Lys, and Arg on key parameters involved in muscle cell functionality. A high-fat diet/low streptozotocin dose method was utilized to induce type 2 diabetes mellitus in rats. Rats were fed the high-fat diet supplemented with HMB and amino acids (HAas), with slow digestive carbohydrates (SDCs), and with a mixture of SDCs and HAas for one month. The expression of creatine kinase (**a**) and GLUT4 transporter (**b**) and the activation of Akt (**c**) were measured in the skeletal muscle lysates. Data are expressed as mean ± SEM (*n* = 4). * *p* < 0.05 vs. rats fed the HF/STZ diet; # *p* < 0.05 vs. high-fat diet/streptozotocin rats.

At the end of the experimental period, gastrocnemius muscles from the different experimental groups were isolated, and the expression of key parameters involved in the muscle cell functionality was assayed in the muscle extracts, as described in the experimental section (Figure 7). In the animals fed the high-fat diet with rapid digestive carbohydrates supplemented with the amino acid mixture and HMB (HF+HAas/STZ), a significant increase in the creatine kinase (Figure 7a), GLUT4 transporter (Figure 7b), and Akt phosphorylation (Figure 7c) compared with the HF/STZ group was detected. However, the group supplemented with SDCs, Lys, Arg, and HMB showed a significantly higher positive effect on the creatine kinase expression, GLUT4 transporter amount, and Akt phosphorylation.

## 4. Discussion

The present study aimed to evaluate the potential benefits of supplementation with HMB, Lys, and Arg to break the vicious cycle of muscle loss and diabetes progression [38]. By targeting multiple pathways involved in muscle protein synthesis and glucose metabolism, this combination of nutrients may work synergically to improve glucose uptake by muscle, stimulate muscle protein synthesis, reduce muscle protein degradation, and counteract insulin resistance. By exploring the unique characteristics of each ingredient and its potential synergy, we seek to provide a novel nutritional strategy that addresses the tremendous changes in the skeletal muscle, concerning both muscle atrophy and glucose utilization in diabetic patients [39].

Related to protein metabolism, our in vitro study in L6 muscle cells revealed increased net protein synthesis and decreased protein degradation in response to HMB treatment. These results are consistent with our previous research demonstrating that HMB was the metabolite responsible for the leucine-enhancing effect on protein synthesis in muscle cells [32] and that HMB could reduce protein degradation by downregulation of FOXO pathways [31]. Furthermore, Wilson et al. [40] conducted a meta-analysis of randomized controlled trials, and they reported that HMB supplementation significantly increased muscle protein synthesis and attenuated protein degradation in both young and older individuals. Moreover, the review conducted by Sanz-Paris [41] concluded that HMB supplementation improved muscle protein synthesis and reduced muscle protein breakdown in various populations, including older adults and patients with chronic diseases.

When the effect of HMB on glucose uptake was investigated, our study showed that the different HMB concentrations tested decreased glucose uptake in skeletal muscle cells. This was due to a decrease in the total amount of the GLUT4 transporter and its translocation to the membrane at the higher HMB concentration tested. These results are consistent with those of previous preclinical studies that showed that HMB might impair insulin sensitivity. In fact, it has been described in normoglycemic rats that the supplementation of HMB induced an apparent insulin-resistant state and a reduction of muscle GLUT4 content [19,20]. A similar action of HMB was described in preclinical models of diabetes [21]. On the contrary, in a study with animals presenting with insulin resistance, HMB supplementation could rescue the insulin resistance observed [17,18]. This effect of HMB on glucose homeostasis has also been observed in older people [42].

The molecular mechanisms underlying the effects of HMB were investigated, considering the lack of a consensus on the outcome of HMB on glycemic control by skeletal muscle. Some of the studies point out that the inhibition of glucose uptake could be due to an overstimulation of the mTOR signaling pathways [43]. Given these data, the phosphorylation of IRS-1, a critical component of the insulin signaling pathway, was analyzed for multiple residues. HMB increased the phosphorylation of IRS-1 at Ser302, activating the insulin pathway, but this response decreased at higher doses. An increase in phosphorylation of inhibitory residues of IRS-1 was observed with 50 µM HMB, together with a decrease in GLUT4 translocation to the plasma membrane, possibly due to the lower phosphorylation of the AS160 protein [44]. In short, these data could suggest that HMB modulates the insulin signaling pathway and GLUT4 translocation in a dose-dependent manner: lower doses enhance insulin signaling, whereas higher doses may have inhibitory effects.

To extend the findings of cell culture experiments to an animal model to assess the effects of HMB supplementation on insulin sensitivity, a euglycemic–hyperinsulinemic clamp technique was performed. The clamp, known as the gold-standard technique for measuring insulin sensitivity, allows for a direct and accurate assessment of insulin response [36,45]. Widely used in preclinical and clinical research, it provides crucial insights into metabolic function and glycemic control. In our study, a high-fat rat model of insulin resistance was utilized, and the rats were divided into different dietary groups with or without HMB supplementation. Consistent with previous findings [46], the results demonstrated that feeding a high-fat diet led to the development of insulin resistance in the rats. Interestingly, when the high-fat diet was supplemented with HMB, no significant differences in insulin sensitivity were observed compared to the group fed the high-fat diet alone. These results align with the outcomes obtained from the cell culture experiments, suggesting a lack of improvement in insulin sensitivity upon HMB supplementation.

Therefore, although HMB is responsible for maximizing the net protein balance, it might not offer any additional benefit at the level of glucose metabolism in skeletal muscle. Therefore, combining HMB with other ingredients that can improve skeletal muscle glucose utilization and, thus, potentiate its action on the diabetic muscle could be an effective strategy for preventing the onset of diabetes-related muscle atrophy and mitigating its progression. Oral Arg administration has also shown improved hepatic and peripheral insulin sensitivity [47], and it has been associated with decreased oxidative stress and enhanced cellular antioxidant regulation [48]. Long-term Arg therapy has been shown to prevent or delay the onset of T2DM and maintain long-lasting effects on diabetes incidence, insulin secretion, oxidative stress, and endothelial function in high-risk individuals [49]. These findings suggest the potential of Arg as a complementary approach in T2DM management.

Another possible protective ingredient against diabetic progression is Lys. In individuals with diabetes, Lys supplementation has been shown to inhibit the non-enzymatic glycation of proteins, contributing to maintaining protein structure and function [50]. Studies have demonstrated that Lys supplementation decreases fasting blood glucose and inhibits the glycation of fibrinogen, a plasma glycoprotein involved in the homeostasis system [51]. Clinical trials have shown that Lys supplementation improves protein function and reduces markers of glycemic control, such as HbA1c and fructosamine, in individuals with T2DM [52].

Our findings in muscle cell cultures revealed that Lys and Arg had a significant effect on enhancing glucose uptake. Subsequently, various combinations of Lys and Arg were explored, and all tested concentrations were found to improve glucose uptake. Based on these results, a combination of 10 mM Lys and 5 mM Arg was selected to analyze its synergistic effect with different concentrations of HMB.

When glucose uptake was investigated, the data demonstrated that supplementation with the amino acids combination effectively counteracted the impact of HMB across a range of HMB concentrations (12.5 to 50 µM). Notably, at a concentration of 12.5 µM HMB combined with Lys and Arg, a significant increase in glucose uptake was observed, suggesting a potential synergistic effect.

Moreover, we examined the individual effects of Lys and Arg on protein synthesis. Interestingly, while Lys barely modifies protein synthesis, Arg demonstrated a moderate increase. Remarkably, when the amino acid combination was tested in the presence of increasing concentrations of HMB, an enhancer effect on protein synthesis was observed at 25 µM HMB. This result would agree with previous studies where the nutritional supplementation of the combination of HMB, Lys, and Arg in elderly healthy people increased protein turnover and lean tissue [53,54], as well as showed a beneficial effect on muscle functionality [55].

Therefore, these findings indicate that HMB at lower doses, and especially when combined with Lys and Arg, not only has no negative effect on glucose uptake but has a clear positive effect on it. Importantly, this approach preserves the positive effects of HMB in protein turnover (by increasing protein synthesis and reducing protein degradation). To better understand the underlying mechanisms, we evaluated crucial parameters involved in muscle glucose uptake. Consistent with the glucose uptake data, Lys and the combination of Arg and Lys significantly increased the total amount of GLUT4. Importantly, even in the presence of 25 µM HMB, the increase in GLUT4 levels persisted. Furthermore, when we analyzed the expression of GLUT4 at the plasma membrane, supplementation with the amino acid mixture resulted in a significant increase in GLUT4 translocation to the plasma membrane, particularly in cells incubated with up to 25 µM HMB. These findings support the positive effect of the amino acid combination with HMB on glucose uptake.

Lastly, considering the molecular mechanisms underlying the supplementation of HMB alone that we described above, the phosphorylation status of IRS-1 at residues associated with the inhibition of insulin signaling was investigated. Our results indicated that HMB-mediated phosphorylation at these inhibitory residues decreased upon incubation with the amino acid mixture. Consequently, the concomitant phosphorylation of Akt, a downstream target of IRS-1, was sustained in the presence or absence of HMB.

This synergistic positive effect of the combination of Lys, Arg, and HMB was also observed when cells were grown to mimic a state of insulin resistance. In the presence of palmitate, which impairs glucose uptake [29], supplementation with individual amino acids, as well as HMB in combination with the amino acid mixture, exhibited significant improvements in glucose uptake. This suggests that the supplementation of specific amino acids, particularly in conjunction with HMB, can reduce the effect on insulin resistance and enhance glucose uptake in muscle cells.

Furthermore, the effects of amino acid supplementation on protein turnover were assessed in the insulin-resistant cell model. It is well-known that insulin resistance leads to decreased protein synthesis [11], but our results demonstrate that HMB supplementation, either alone or in combination with amino acids, can increase protein synthesis compared to cells treated with palmitate alone. Additionally, the transcriptional activity of the ubiquitin promoter, which reflects protein degradation, was examined. In cells pre-incubated with palmitate, the transcription-dependent activity of the ubiquitin promoter was increased, suggesting enhanced protein degradation. However, all the treatments, including HMB alone and the combination of HMB with amino acids, significantly decreased this transcriptional activity. These findings suggest that HMB, Lys, and Arg supplementation maximizes the net protein balance, mitigating the changes in skeletal muscle protein turnover associated with insulin resistance.

To validate and extend these positive results, the effect of the HMB, Lys, and Arg supplementation was analyzed in a well-established rat model of diabetes [37]. Additionally, the concept of slow digestible carbohydrates (SDCs) was included in this study to maximize the beneficial effect of specific nutritional formulations for people with diabetes. SDCs are carbohydrates that are digested gradually, allowing for a steady release of glucose, which prevents postprandial glucose spikes and, consequently, lowers insulin requirement [56]. Moreover, the positive effect of SDCs on glycemic control in patients with diabetes has been broadly described [57,58].

One notable finding was the significant increase in body weight observed in the group fed the SDC diet supplemented with HMB, Lys, and Arg compared to the other experimental groups. This increase in body weight was accompanied by an increase in lean body mass. In our animal model, the diabetic group (HF/STZ) lost more than half of LBM due to the effects of the diabetes progression. Interestingly, the group fed the SDC diet supplemented with HMB, Lys, and Arg was the group that lost significantly less LBM, losing less than 25% of their LBM. These findings were particularly interesting in the context of diabetes, as diabetics often experience muscle mass loss [5,7]. For instance, in the Health ABC study, the impact of T2DM on the changes in body composition was analyzed [2,5,6]. In this cohort of 2675 participants, patients with diabetes were observed to have high LBM loss associated with body weight changes (approximately 32% in undiagnosed diabetic patients and 36% in diagnosed diabetic patients) in comparison with participants without diabetes. Our finding is particularly remarkable in the context of diabetes, suggesting that the combination of HMB, Lys, and Arg may promote protection from the deleterious effect of diabetes progression on muscle health, especially in malnourished diabetes patients.

The next important point of regulation to break the vicious cycle that we have discussed before is glycemic control. There is strong evidence showing that an improvement in muscle mass and/or muscle strength improves insulin sensitivity and prevents insulin resistance [59,60,61,62]. In our model, a significant decrease in glycemia was observed in the group fed the SDC diet supplemented with HMB, Lys, and Arg, together with a significantly lower insulin resistance index and a concomitant and significant increase in the insulin sensitivity index. This finding suggests that the combination of HMB, Lys, Arg, and SDCs has a positive impact on glucose control and insulin sensitivity. The molecular analysis of gastrocnemius muscle further supports these beneficial effects of HMB, Lys, and Arg on insulin resistance. The lower GLUT4 expression and Akt phosphorylation in the diabetic control group reflect impaired glucose uptake and insulin signaling in the muscle. In contrast, the group fed a diet supplemented with HMB, Lys, and Arg groups, regardless of the presence of SDCs in the diet, showed improvements in these parameters. These results suggested that the combination of HMB, amino acids, and slow digestive carbohydrates promotes glucose uptake and insulin signaling in the muscle.

The better regulation of glucose homeostasis was also reflected in the effect of diet on glycosylated hemoglobin levels. In patients with diabetes, sarcopenia has been positively associated with HbA1c levels. In the MUSCLE-DM study, patients with a reduction of about 1% in HbA1c showed a significant increase in skeletal muscle index and gait speed [60]. In our model, dietary supplementation with HMB, Lys, Arg, and SDCs reduced the percentage of HbA1c by approximately 2.5% compared to the diabetic control group. These data are relevant if one takes into account that HbA1c levels not only reflect poor glycemic control in patients with type 2 diabetes and may indicate a risk of sarcopenia but also correlate with the risk of long-term diabetic complications [63,64].

In terms of cardiovascular risk, the HF+HAas+SDC/STZ group showed a significant decrease in triglyceride and cholesterol levels compared to the other experimental groups. Elevated levels of triglycerides and cholesterol are associated with an increased risk of cardiovascular complications in individuals with diabetes [65]. The reduction in these risk factors suggests that the combination of HMB, amino acids, and SDCs may have protective effects on cardiovascular health in the context of diabetes.

## 5. Limitations of the Study

Although the results obtained in this article are relevant for the design of strategies to treat diabetes and sarcopenia, there are some limitations in the study. First, only an animal model of diabetes was used. In addition, the model used, based on the injection of STZ, limited the extension time of the study. Long-term studies to address the effects of the ingredients on the long-term complications of diabetes would be needed. Finally, in the study, slow digestive carbohydrates were used, but it would be interesting to study the effects of the ingredients associated with other carbohydrates to be used in other pathologies that present sarcopenia not associated with diabetes. Therefore, additional research is needed to translate the beneficial outcomes of this preclinical study into clinical practice.

## 6. Conclusions

Overall, our findings highlight the potential benefits of using HMB in combination with Lys and Arg in specialized nutritional formulas, which include SDCs, on diabetes management. This combination appears to exert positive metabolic effects, breaking the vicious cycle between sarcopenia and diabetes by preserving skeletal muscle mass, improving muscle glucose uptake, and attenuating the underlying insulin resistance. These metabolic-related changes in skeletal muscle may also have implications for the progression of associated diabetes complications. In our view, given the importance of maintaining skeletal muscle mass and function to normalize the overall metabolism in type 2 diabetes, the present preclinical study (in vitro and animal T2DM models) provides evidence supporting the use of HMB and Lys–Arg mixture as a nutritional approach to preserve healthy skeletal muscle in individuals with T2DM by normalizing signaling pathways that were impaired in diabetes. These findings may contribute to the development of strategies for optimizing the beneficial effects of HMB in the context of muscle insulin resistance and provide a foundation for future research in this area.

## Data Availability

The raw data supporting the conclusions of this article will be made available by the authors.

## Supplementary Materials

The following supporting information can be downloaded at https://www.mdpi.com/article/10.3390/nu15224706/s1, Figure S1. Effects of HMB on the phosphorylative status of the insulin signaling pathway; Figure S2. Cardiovascular risk in the different diabetes experimental groups; Table S1. Composition of Diets for the Clamp Study; Table S2 Composition of Diets for the models of diabetes; Table S3. Body weight and daily food intake of the experimental groups over time.
