# Supplementary material for: Arginine and Lysine Supplementation Potentiates the Beneficial β-Hydroxy ß-Methyl Butyrate (HMB) Effects on Skeletal Muscle in a Rat Model of Diabetes
Source: Nutrients. 2023 Nov 7;15(22):4706. doi: 10.3390/nu15224706 (PMC10674618; doi:10.3390/nu15224706)
Supplement: Supplementary file 1 [file nutrients-15-04706-s001.zip › nutrients-2696960-supplementary.pdf]

Article

Arginine and Lysine Supplementation Potentiates the Beneficial β-Hydroxy ß-Methyl Butyrate (HMB) Effects on Skeletal Muscle in a Rat Model of Diabetes

Manuel Manzano [1,†], María D. Girón [2,†], Rafael Salto [2,*], Chiara Burgio [2], Antonio Reinoso [2], Elena Cabrera [2], Ricardo Rueda [1] and Jose M. López-Pedrosa [1]

[1] Abbott Nutrition R&D, E18004 Granada, Spain; manuel.manzano@abbott.com (M.M.); ricardo.rueda@abbott.com (R.R.); jose.m.lopez@abbott.com (J.M.L.-P.)
[2] Department of Biochemistry and Molecular Biology II, School of Pharmacy, University of Granada, E18071 Granada, Spain; mgiron@ugr.es (M.D.G.); chiaraburgio@ugr.es (C.B.); areinoso@ugr.es (A.R.); elenacc202@gmail.com (E.C.)
* Correspondence: rsalto@ugr.es
† These authors contributed equally to this work.

Electronic Supplementary Materials

1.- **Supplemental Material Table S1** *Composition of Diets for the Clamp Study.*

2.- **Supplemental Material Table S2** *Composition of Diets for the models of diabetes.*

3. **Supplemental Material Figure S1.** *Effects of HMB on the phosphorylative status of the insulin signaling pathway.*

4. **Supplemental Material Table S3.** *Body weight and daily food intake of the experimental groups over time.*

5. **Supplemental Material Figure S2.** *Cardiovascular risk in the different diabetes experimental groups.*

1. Supplemental Material Table S1. Composition of diets for the clamp study

Macronutrients	AIN93M	HF	HF-H
**CHO** *(g/100g diet)*	75.72	44.70	44.70
**Rapid Digesting Simple Sugar** *(g/100g diet)*	10.21	16.90	16.90
**Rapid Digesting Complex Sugar** *(g/100g diet)*	60.39	16.63	16.63
**Indigestible Fiber** *(g/100g diet)*	5.11	11.18	11.18
**Protein** *(g/100 g diet)*	12.58	24.19	24.19
**l-lysine** *(g/100g diet)*	1.00	2.00	2.00
**l-arginine** *(g/100 g diet)*	0.50	1.00	1.00
**HMB** *(g/100 g diet)*			0.78
**Fat** *(g/100 g diet)*	4.00	20.00	20.00
**Soy oil** *(g/100 g diet)*	4.00		
**Lard fat** *(g/100 g diet)*		20.00	20.00
**Energy** *(Kcalories/100 g diet)*	379	433	433

HF: high-fat diet; H: beta-hydroxy beta-methylbutyrate.

2. **Supplemental Material Table S2. Composition of diets for models of diabetes**

Macronutrients	HF	HF+HAas	HF+SDC	HF+HAas+SDC
**CHO** *(g/100g diet)*	44.70	44.70	44.70	44.70
**Rapid Digesting Simple Sugar** *(g/100g diet)*	16.90	16.90		
**Slow Digesting Simple Sugar** *(g/100g diet)*			12.96	12.96
**Rapid Digesting Complex Sugar** *(g/100g diet)*	16.63	16.63	15.78	15.78
**Polyols** *(g/100g diet)*			4.11	4.11
**Resistant Starch** *(g/100g diet)*			6.71	6.71
**Indigestible Fiber** *(g/100g diet)*	11.18	11.18	5.14	5.14
**Protein** *(g/100 g diet)*	24.19	24.19	24.19	24.19
**l-lysine** *(g/100g diet)*	2.00	3.00	2.00	3.00
**l-arginine** *(g/100 g diet)*	1.00	1.50	1.00	1.50
**HMB** *(g/100 g diet)*		0.78		0.78
**Fat** *(g/100 g diet)*	20.00	20.00	20.00	20.00
**Soy oil** *(g/100 g diet)*				
**Lard fat** *(g/100 g diet)*	20.00	20.00	20.00	20.00
**Energy** *(Kcalories/100 g diet)*	433	433	433	433

HF: high-fat diet; H: beta-hydroxy beta-methyl-butyrate; SDC: slow digestive carbohydrates; HAas: mixture de HMB, Lys, and Arg.

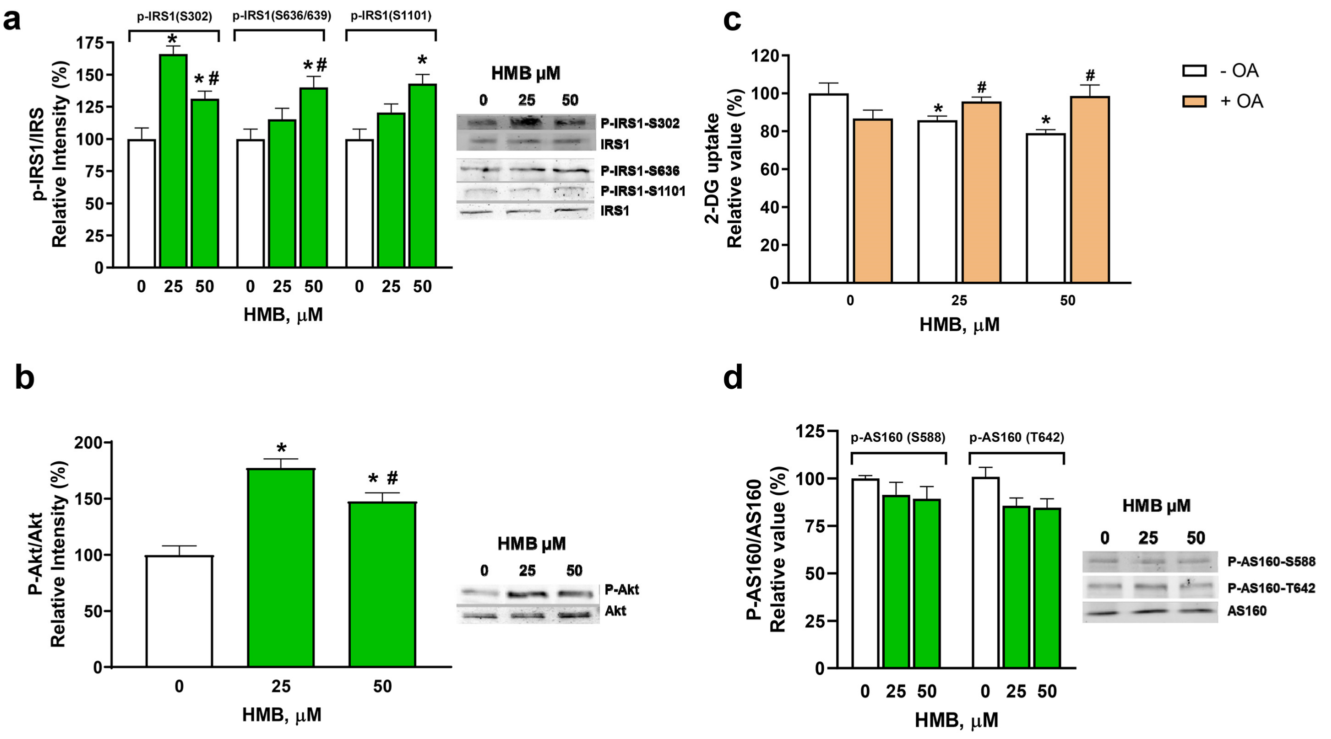

2. Supplemental Material Figure S1. Effects of HMB on the phosphorylative status of the insulin signaling pathway. L6 myotubes were incubated with HMB (0-50 μM) for 30 min and the phosphorylation of IRS-1 at several residues (a) and of Akt (b) was measured. (c) and (d) show the involvement of AS160 in the translocation of GLUT4 in response to HMB. (c) 2-DG uptake was measured in cells incubated in the presence or absence of 1mM Okadaic Acid (OA) for 2 h. (d) The effects of HMB (30 min) were measured on the phosphorylation of the insulin-responsive AS160 residues. Data were expressed as mean ± SEM (n=4). * p<0.05 vs cells incubated in the absence of effectors; in graphs a and b, # p<0.05 vs HMB 25 μM incubated cells. In graph c, # p<0.05 vs OA incubated cells.

3. Supplemental Material Table S3. Body weight and daily food intake of the experimental groups over time.

	HF/STZ	HF+HAas/STZ	HF+SDC/STZ	HF+HAas+SDC/STZ
Body weight before STZ injection *(g)*	458.9 ± 12.8	445.9 ± 11.4	431.5 ± 8.1	399.2 ± 11.2*#$
Body weight at the end of the study *(g)*	413.8 ± 13.7	416.6 ± 21.3	389.6 ± 39.2	412.1 ± 15.4
Daily food intake before STZ injection *(kcal/day·kg bw)*	147.6 ± 3.8	147.5 ± 2.0	122.5 ± 2.1*#	117.3 ± 2.9*#
Daily food intake after STZ injection *(kcal/day·kg bw)*	237.9 ± 13.3	274.1 ± 21.5	228.6 ± 7.46	239.5 ± 8.4

Data expressed as mean±SEM. A significant difference (p<0.05) among groups (*) vs HF/STZ group, (#) vs HF+HAas/STZ group, ($) vs HF+SDC/STZ group as shown analysis of Student`s t-test.

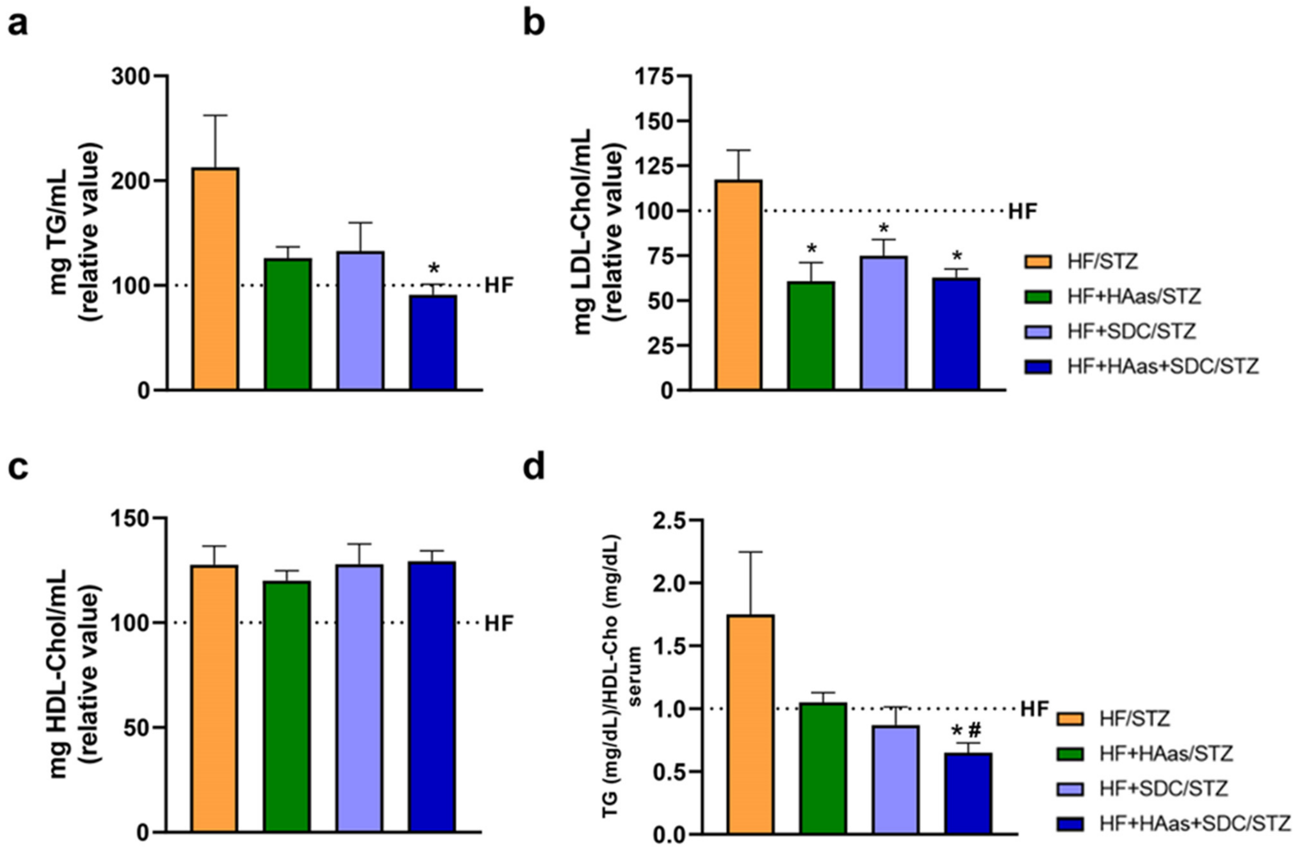

4. Supplemental Material Figure S2. *Cardiovascular risk in the different diabetes experimental groups.* Relative values of triacylglycerols (TG) (a), LDL Cholesterol (b), and HDL Cholesterol (c) are shown. Values are referred to those before the diabetes induction by STZ injection (HF). Cardiovascular risk (d) was calculated based on the above determinations. Data were expressed as mean ± SEM (n=10). * $p<0.05$ vs HF/STZ. In graph d, # $p<0.05$ vs HF+HAas/STZ group.
